# The application of PTR-MS and non-targeted analysis to characterize VOCs emitted from a plastic recycling facility fire

**DOI:** 10.1038/s41370-024-00681-y

**Published:** 2024-05-06

**Authors:** Eva C. M. Vitucci, Oladayo Oladeji, Albert A. Presto, Carolyn L. Cannon, Natalie M. Johnson

**Affiliations:** 1 Department of Environmental and Occupational Health, Interdisciplinary Faculty of Toxicology, Texas A&M University, College Station, TX 77843 USA; 2 Department of Mechanical Engineering, Carnegie Mellon University, Pittsburgh, PA 15213 USA; 3 Department of Microbial Pathogenesis and Immunology, School of Medicine, Texas A&M University, Bryan, TX 77807 USA

**Keywords:** Air pollution, Analytical methods, Volatile organic compounds, Inhalation exposure, Disaster

## Abstract

**Background:**

On April 11th, 2023, the My Way Trading (MWT) recycling facility in Richmond, Indiana caught fire, mandating the evacuation of local residents and necessitating the U.S. Environmental Protection Agency (EPA) to conduct air monitoring. The EPA detected elevated levels of plastic combustion-related air pollutants, including hydrogen cyanide and benzene.

**Objective:**

We aimed to identify these and other volatile organic compounds (VOCs) present as well as to identify the potential hazard of each compound for various human health effects.

**Methods:**

To identify the VOCs, we conducted air monitoring at sites within and bordering the evacuation zone using proton transfer reaction mass spectrometry (PTR-MS) and non-targeted analysis (NTA). To facilitate risk assessment of the emitted VOCs, we used the EPA Hazard Comparison Dashboard.

**Results:**

We identified 46 VOCs, within and outside the evacuation zone, with average detection levels above local background levels measured in Middletown, OH. Levels of hydrogen cyanide and 4 other VOCs were at least 1.8-fold higher near the incidence site in comparison to background levels and displayed unique temporal and spatial patterns. The 46 VOCs identified had the highest hazardous potential for eye and skin irritation, with approximately 45% and 39%, respectively, of the VOCs classified as high and very high hazards for these endpoints. Notably, all detected VOC levels were below the hazard thresholds set for single VOC exposures; however, hazard thresholds for exposure to VOC mixtures are currently unclear.

**Impact:**

This study serves as a proof-of-concept that PTR-MS coupled with NTA can facilitate rapid identification and hazard assessment of VOCs emitted following anthropogenic disasters. Furthermore, it demonstrates that this approach may augment future disaster responses to quantify additional VOCs present in complex combustion mixtures.

## Introduction

Large anthropogenic and natural disasters release thousands of chemicals into the environment every year [1–3]. Smaller scale events, such as fires at recycling plants, are commonly overlooked contributors and are increasing in frequency throughout the U.S. [4–6]. Recycling plants and warehouses contain large amounts of flammable material resulting in persistent, high temperature fires [7, 8]. Consequently, a plethora of harmful toxics, such as particulate matter and volatile organic compounds (VOC) are released into the local communities [9–12]. Due to the variety of material stored at recycling plants, the composition of the burned substrates is commonly unknown, limiting the ability to accurately predict VOC combustion products and emissions. Thus, there is a significant need for strategies that can rapidly identify VOC emissions and assess the health risks they impose on the local communities following these fires [5].

Traditional field-deployable analytical approaches to detect air toxics following disasters are frequently limited by the sensitivity of the instrument, the time resolution of sampling, and the ability to characterize a broad range of pollutants [13, 14]. Mass spectrometry coupled with non-targeted analysis (NTA) is an emerging field that rapidly identifies unknown compounds independent of prior knowledge of the initial substrate [1, 5, 14–16]. Using mock disaster scenarios, Sloop et al. and Phillips et al. previously suggested how NTA can be incorporated into disaster response scenarios [1, 5]. They and others highlight key metrics such as analysis speed, confidence in chemical identification, and incorporation of hazard assessment that can enhance the beneficial impact of NTA in disaster scenarios [1, 5, 14, 17]. Here, we build upon this work and demonstrate a novel application of this metric-based workflow to detect emitted VOCs following a real disaster scenario.

On Tuesday April 11th, 2023, the My Way Trading (MWT) recycling warehouses containing recycled plastics and other hazardous material caught fire in Richmond, Indiana [18]. The MWT fire was active for 5 days, April 11th – April 16th [18], and required approximately 2000 residents that lived within 0.5 miles of the warehouses to evacuate. Given the potential release of hazardous combustion products into the community, the U.S. Environmental Protection Agency (EPA) performed continuous air monitoring for predicted combustion products of plastics within and outside of the evacuation zone. Within the evacuation zone the EPA detected elevated levels of particulate matter and the hazardous VOCs, benzene, carbon monoxide, chlorine, and hydrogen cyanide [18, 19]. To complement the EPA air monitoring data, we conducted stationary air monitoring using a highly sensitive proton transfer reaction time-of-flight mass spectrometer (PTR-ToF-MS) to characterize the emitted VOCs at varying distances within and outside of the evacuation zone [20]. This approach offered complementary evidence, and the ability to quickly adapt location based on wind speed/direction changes or local policy updates, in comparison to traditional stationary air monitors set at specific point locations. Moreover, our instrumentation (PTR-ToF-MS) offered highly sensitive and resolved VOC data to discriminate complex mixtures following this emergency event.

As the MWT warehouses contained large mixtures of unknown substrates, we performed NTA to generate a comprehensive list of the emitted VOCs and identify the VOCs with the largest increases in relative concentration. We then evaluated the emission relationships between the VOC within and bordering the evacuation zone and generated a hazard assessment of identified VOCs using the EPA Hazard Comparison Dashboard [5, 21–23] to evaluate the potential human health risks associated with the chemical exposures. We illustrate that coupling PTR-MS with NTA is a rapid and viable approach to generate a comprehensive list of emitted VOCs and evaluate their hazard potential.

## Materials and methods

### Instrumentation

Air monitoring measurements were carried out at three different locations using the Texas A&M University Mobile Responding to Air Pollution in Disasters (mRAPiD) van. The mRAPiD van is fitted with a sample inlet that extends upward, approximately 3.5 m from the roof of the van. In brief, 0.5” outer diameter Teflon tubing connects the external sample inlet to a mechanical backing pump, which pulls ambient air through the tubing line. Ambient air is pulled from this tubing line through a 1/16” outer diameter PEEK tubing into the PTR-ToF-MS 4000 (IONICON, Inc.) trace gas analyzer at a flow rate of 200 standard cubic centimeters per minute (sccm). Analysis of VOCs present in the air sample occurred every 1 s, with hydronium (H_3_O^+^) as the ion source. Chemical species with a higher proton affinity than water are detectable when using (H_3_O^+^) as the ion source, resulting in the ability to detect a variety of atmospheric VOCs while limiting the detection of smaller aliphatic molecules that would otherwise dominate the signal. Full PTR-ToF operations and analysis details can be found in the supplemental information and in Oladeji et al. (Supplementary Method S1 and Supplementary Table S5) [14].

### Sampling strategy

**Fig. 1:**
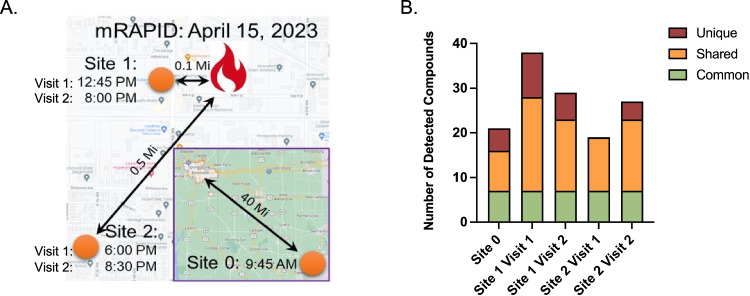
Stationary air monitoring detects sampling site-specific emissions. **A** Map of stationary air monitoring sampling sites. The location of the My Way Trading warehouse fire is depicted by the flame icon. Site 1 visits are within the evacuation zone and Site 2 visits are outside the evacuation zone. **B** Number of compounds detected at each sampling site visit. “*Common*” compounds are compounds detected at all sampling sites and visits. “*Shared*” compounds are compounds detected at multiple, but not all, sampling sites and visits. “*Unique*” compounds are compounds detected at only one sampling site visit.

Air monitoring was conducted at the following 3 sites: Site 0 (Middletown, OH; local background, 40 miles from the incidence site), Site 1 (0.1 miles from the incidence site; within the evacuation zone), and Site 2 (0.5 miles from the incidence site; bordering the evacuation zone) (Fig. 1A). Site 0 was selected to collect background VOC levels to help discriminate if VOC levels detected closer to the incidence site were elevated or decreased relative to “typical” background areas. Site 1 and Site 2 were selected to generate measurements at locations near and bordering the evacuation zone to help inform the potential impact of local emissions throughout the community. Sampling at Site 1 and Site 2 was conducted twice (Visit 1 and Visit 2) within the same day to capture any temporal differences in VOC emissions that could occur as the MWT warehouse fire continued to smolder. Stationary air sampling at these discrete locations while parked was employed as this was still an active emergency event with a clear evacuation boundary. All stationary sampling was conducted continuously for 30-minutes at each site and occurred between 9:45 am and 9:00 pm on April 15^th^, 2023. Weather data during each sampling period was collected from local weather monitoring stations, ID#KOHMIDDL47 and ID#KINRICHM45, in Middletown, OH and Richmond, IN, respectively (Supplementary Table S4). Weather data was accessed through Weather Underground, https://www.wunderground.com/.

### VOC identification

Identification and quantification of VOCs was conducted using the standard software package, IONICON PTR-MS Viewer analysis software, Version 3.4.4, provided by IONICON [24]. Individual compounds detected by the ion detector at the default minimum of 5 counts per second (cps), were considered for analysis. Any compound below this set threshold value was considered noise and excluded from analysis. To identify the VOCs present during the sampling periods the spectrum of sampled ambient air was compared to the IONICON compound database within the PTR-MS Viewer software. For each spectrum a list of suggested compounds was generated by the PTR-MS Viewer software based on the mass of the selected spectral peak. A “match probability” percentile was further calculated by the PTR-MS Viewer software for each predicted compound based off the isotope distribution and the exact mass to charge ratio (*m/z*). Suggested compounds with a “match probability” to the spectrum of at least 90% were selected for subsequent analyses and listed in Supplementary Table 2.

### VOC quantification

Ion signals detected by the PTR-TOF-MS were converted to final VOC concentrations in parts per billion volume (ppbv) in the PTR-MS Viewer software through the application of the following equation:

$$(d[{{XH}}^{+}])/{dt}=k[H3O][X]$$

Quantification of the VOCs detected using NTA was determined assuming the reaction time between each analyte, “X”, and H3O is constant (as is in the current sampling strategy) and by using the default rate constant (k) of 2 × 10^−9 cm^−3^ molecule^−1^ s^−1^ for each analyte as a known k value was not available for all of the detected analytes. Notably, this default value has been used widely in atmospheric chemistry literature when *k* is not directly available and is capable of measuring uncalibrated compounds with an accuracy of ±30% [25]. A value of “0” was used to replace any non-detected (below instrument limit of detection) measurement of a compound during the sampling period to generate a conservative average concentration of each compound. Of this initial list, only compounds with estimated average VOC concentrations higher than the background sample were included for subsequent analyses. Note, the compound C5H9, commonly referred to as 1,3-Butadiene was excluded from analyses due to poor resolution of this compound from background humidity when using H3O^+^ as the PTR-MS ion source. In addition, as the compounds CO_2_, NO_2_, and N_2_O (commonly referred to as carbon dioxide, nitrogen dioxide, and nitrous oxide, respectively) are not considered VOCs and as it is difficult to discern if their detected signal is the result of fragmentation from other detected analytes, these compounds were excluded from subsequent analysis. Together, these filters resulted in a curated list of 46 detected VOCs (Supplementary Table 2). to be carried forward in analyses.

Quantification of the VOCs detected using targeted analysis (benzene and toluene) was also determined assuming the reaction time between each analyte, “X”, and H3O is constant (as is in the current sampling strategy) and by using the default rate constant (k) of 2 × 10^−9 cm^−3^ molecule^−1^ s^−1^ for each analyte. The calculated concentrations for benzene and toluene were then multiplied by a predetermined calibration factor (1.83 and 2.69), derived from a calibration gas of known concentration, to account for instrument-specific variability in quantifying VOC concentrations. A value of “0” was used to replace any non-detected (below instrument limit of detection) measurements of benzene or toluene during the sampling period to generate a conservative average concentration of each compound. To identify significant relationships (*p*_adj_ < 0.05) between benzene levels across sampling sites, a Kruskal–Wallis test followed by a Dunn’s multiple comparison test was used in Prism10 Version 10.0.3.

### VOC correlation assessment

To identify significant relationships between the VOCs emitted across the site visits, a correlation matrix was computed using the estimated, average concentration of the curated VOCs in RStudio Version 2023.06.0 + 421. The Spearman’s rank correlation analysis was used to identify significant relationships (*p* < 0.05).

### VOC hazard assessment

To generate a hazard assessment of the detected VOCs, the predicted chemicals associated with each formula in the curated list were first input into the EPA CompTox Chemicals Dashboard (from here on referred to as the Dashboard) [21, 22] to validate their identity using the “batch search” function (10.1021/acs.jcim.0c01273) [21, 22]. Any chemical that was not identified in the Dashboard was excluded from further analysis. The remaining list of predicted compounds was then input into the Hazard Comparison Module to provide data which could be used for the hazard assessment of each compound for human health effects (Supplementary Table 4). It should be noted that ecotoxicological toxicity effects are also available (as seen in Supplementary Table 4), but these were not applied in this study.

## Results

### Overview of sampling and detection

Non-targeted analysis of stationary air monitoring detected 59 unique compounds VOCs across all sampling site visits (Supplementary Table S1 and Fig. 1A). The number and combinations of compounds detected between sites and site visits varied, with 21 at Site 0, 38 at Site 1 (Visit 1), 29 at Site 1 (Visit 2), 19 at Site 2 (Visit 1), and 27 at Site 2 (Visit 2) (Fig. 1B). More compounds were consistently detected at Site 1 on both visits, 0.1 miles from the MWT warehouse fire and within the evacuation zone. In addition to higher numbers of total compounds detected, the highest number of unique compounds, i.e., compounds not detected at any other visit, were also detected at the Site 1 visits, with only 3 unique compounds at Site 0, 10 and 6 unique compounds at Site 1 (visits 1 and 2, respectively) and 0 and 4 unique compounds at Site 2 (visits 1 and 2, respectively) (Fig. 1B).

### Targeted analysis of benzene

**Fig. 2:**
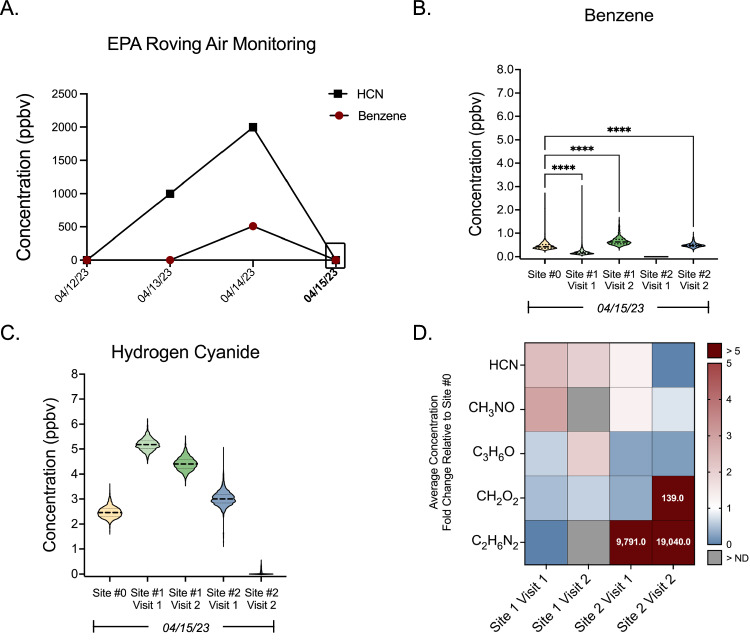
Targeted and non-targeted analysis identify elevated VOCs within and outside the evacuation zone. **A** EPA targeted analysis of benzene and hydrogen cyanide (HCN) within the evacuation zone between April 12–15th, 2023. **B** Targeted detection levels of benzene across sampling site visits on April 15th, 2023. **C** Non-targeted estimated detection levels of hydrogen cyanide across sampling site visits on April 15th, 2023. **D** Non-targeted analysis of VOCs within and outside the evacuation zone with estimated average levels at least 1.8-fold higher than background (site 0) levels. The numerical fold change value is shown for compounds that exceeded a 5-fold increase.

Following the MWT warehouse fire, EPA “Roving Air Monitoring” detected elevated levels of benzene, yet below levels of health concern, within the evacuation zone (Fig. 2A) [18]. Consistent with EPA sampling, we also detected levels of benzene within the evacuation zone (Fig. 2A, B). Targeted analysis of benzene resulted in average and maximum concentrations, respectively, of 0.46 and 2.68 (Site 0), 0.20 and 3.01 (Site 1 Visit 1), and 0.67 and 1.63 ppbv (Site 1 Visit 2). We also detected levels of benzene bordering the mandated evacuation zone, with average and maximum concentrations of 0.49 and 1.01 ppbv at Site 2 Visit 2 (Fig. 2B). Notably, levels of benzene were significantly different between all sampling sites and visits (*p*_adj_ < 0.0001).

### Non-targeted analysis detected additional elevated VOCs

Of the 59 compounds detected by non-targeted analysis, 46 VOCs were detected at sampling sites with average concentration levels higher than the local rural background (Site 0) during at least one site visit (Supplementary Table S2). Notably, HCN (identified as either hydrogen cyanide or hydrogen isocyanide) was the only compound within this curated list that was also detected by the EPA “Roving Air Monitoring” (Fig. 2A) and consistently elevated between multiple sampling visits. Estimated respective average and maximum HCN concentrations were detected at 2.47 and 3.52 (Site 0), 5.19 and 6.12 (Site 1 Visit 1), 4.42 and 5.42 (Site 1 Visit 2), and 3.02 and 4.96 ppbv (Site 2 Visit 2) (Fig. 2C). Levels of HCN were also significantly different between all sampling sites and visits (*p*_adj_ < 0.0001). NTA also identified HCN and 4 additional compounds (C_3_H_6_O, CH_3_NO (formamide), CH_2_O_2_ (formic acid), and C_2_H_6_N_2_) with estimated detection levels at least 1.8-fold higher than background levels during at least one site visit within or bordering the evacuation zone (Supplementary Table S2 and Fig. 2D). C_2_H_6_N_2_ was identified as either dimethyldiazene or ethanimidamide (CH_3_C(=NH)NH_2_), and C_3_H_6_O could represent the potential chemicals: 1) acetone, 2) methoxyethene, 3) oxetane, 4) propanal, or 5) propylene oxide. HCN, CH_3_NO, and C_3_H_6_O fold changes were the highest amongst the Site 1 visits, with HCN concentrations 2.1- and 1.8-fold higher than background levels at Site 1 Visit 1 and Site 1 Visit 2, respectively. CH_3_NO concentrations were 2.6-fold higher than background levels at Site 1 Visit 1, and C_3_H_6_O concentrations were 1.8-fold higher than background levels at Site 1 Visit 2. Concentrations of C_2_H_6_N_2_ and CH_2_O_2_ were the highest amongst the Site 2 visits. C_2_H_6_N_2_ was 9791- and 19,050-fold higher than background at Site 2 Visit 1 and Site 2 Visit 2, respectively. CH_2_O_2_ was 138-fold higher than background at Site 2 Visit 2. Importantly, for the potential chemicals with known exposure limits, all estimated average detection levels remained within the safe exposure limits. Safe exposure limits for the following chemicals are either inconclusive or do not currently exist: Dimethyldiazene, ethanimidamide, methoxyethene, and oxetane.

### Non-targeted analysis detected VOC emission relationships

**Fig. 3:**
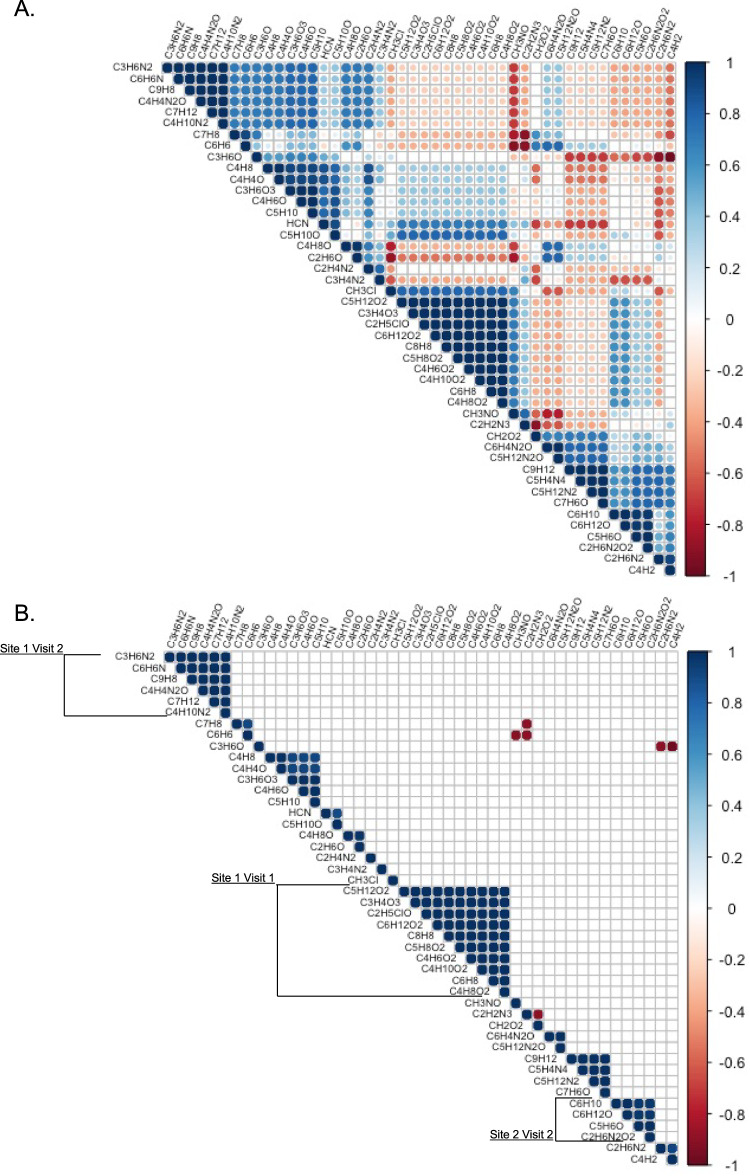
Spearman’s correlation matrix of elevated VOCs within and outside the evacuation zone relative to background levels. **A** Correlation matrix displaying all correlation relationships between the detected compounds. **B** Correlation matrix displaying only the significant (*p* < 0.05) correlation relationships between the detected compounds.

To identify significant relationships between the VOCs emitted across the sampling sites, we generated a correlation matrix using the curated list of VOCs. We detected 5 clusters of at least 4 VOCs that were significantly, positively correlated (Fig. 3A, B). Three of the 5 clusters were composed of VOCs identified at only one sample site visit (Fig. 3B). Notably, benzene and HCN were not significantly correlated with large numbers of other VOCs. Toluene was the only VOC that was significantly, positively correlated with benzene, while CH_3_NO (formamide) and the uncharacterized compound, C_2_H_2_N_3_ were significantly, negatively correlated with benzene. Similar to benzene, HCN was also significantly, positively correlated with only one other VOC, C_5_H_10_O. C_5_H_10_O could represent the following potential chemicals: 1)2-Butanone, 3-methyl-, 2) 2H-Pyran, tetrahydro-, 3) 3-Pentanone, 4) 3-ethoxyprop-1-ene (C2H5OCH2CH=CH2), 5) 1-ethoxyprop-1-ene (C2H5OCH=CHCH3), 6) Furan, tetrahydro-2-methyl-, 7) Pentanal, or 8) trans-CH3CH=CH-OC2H5.

### Hazard assessment of the potential chemicals identifies hazard risk for human health effects

**Fig. 4:**
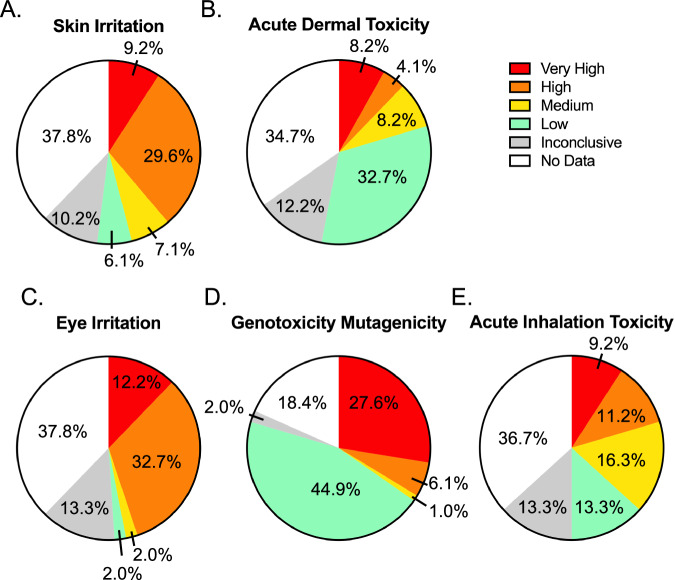
A subset of the hazard assessment of the VOCs detected by non-targeted analysis at sampling sites with average concentration levels higher than the local rural background (Site 0) at any site visit. **A**–**E** The acute human health effects with the highest number of VOCs classified as hazardous, ranging from “very high” to “low” in hazard potential.

To evaluate the potential health hazard of the identified chemicals with average concentration levels higher than the local rural background (Site 0) during at least one site visit we used the EPA Hazard Comparison Module [23] (Supplementary Table S3 and Fig. 4A–E). The EPA Hazard Comparison Module is one of seven modules available on the EPA Cheminformatics Modules website (https://www.epa.gov/chemical-research/cheminformatics). These are proof-of-concept modules to test new ways of delivering data in the CompTox Chemicals Dashboard (CCD) to the community. The Hazard Comparison Module delivers the ToxVal data underlying the CCD as a heat map generated using a trumping scheme rating various human health and ecological toxicity data as Very High, High, Medium, Low and Inconclusive. These ratings are based on the most conservative call from authoritative, screening and QSAR predicted data [23]. The human health effects with the highest number of potential chemicals classified as either a “very high” or “high” hazard were eye irritation (45%) and skin irritation (39%). Genotoxicity mutagenicity had the largest number of potential chemicals classified as “very high” hazards (29%). Acute inhalation toxicity (21%) and acute dermal toxicity (12%) had similar distributions of the number of potential chemicals classified as either a “very high” or “high” hazard. Notably, approximately 30% of the potential chemicals had no published data on eye and skin irritation, acute dermal toxicity, or acute inhalation toxicity.

## Discussion

The MWT warehouse fire was one out of hundreds of recycling facility fires in the U.S. in 2023 [8]. These facilities contain a large mixture and volume of recycled material that can be highly flammable, resulting in persistent, high temperature fires and the release of harmful toxics, such as VOCs. As these facility fires are increasing throughout the U.S. there is a significant need to establish strategies that can sensitively and rapidly detect the emitted VOCs [8]. In addition to identification, there is a need for rapid quantification and health assessment of the detected VOCs to help maintain safe emergency responses and protect the health of local residents. Here we demonstrate that PTR-MS coupled with NTA can facilitate rapid identification and hazard assessment of VOCs emitted following a disaster.

First, we compared spatial variation of benzene and HCN, highly toxic VOCs and common combustion products of plastic materials [9, 11, 26–29]. Air monitoring conducted by the U.S. EPA on April 13th and 14th at sites near the MWT fire detected elevated, but still considered safe, concentrations of benzene and HCN with concentrations ranging between 0.5–2.0 ppm [29, 30]. While benzene and HCN concentrations were below the limit of detection for the air monitoring conducted by the EPA on April 15th, we detected levels of benzene and HCN near the incidence site and bordering the evacuation zone. Notably, we also detected benzene and HCN at our background sampling site, approximately 40 miles away from the incidence site. Concentrations of benzene near the incidence site and bordering the evacuation zone were significantly different than concentrations detected at the background, however, average detection concentrations of benzene across all sampling visits were within national ambient concentrations. Average ambient benzene concentrations ranging between 0.10–1.70 ppb have been detected across the US [31, 32]. While there was lack of a consistent pattern between benzene levels and sampling location, HCN concentrations suggest a pattern between detectable concentrations and proximity to the incidence site. Specifically, HCN concentrations increased as distance to the incidence site decreased. Interestingly, a similar trend in HCN concentrations was detected in a residential area near a gold mining facility, another source of HCN emissions, with concentrations ranging from 0.16 to 8.56 ppbv [33]. Observation of this pattern for HCN, but not benzene, could be due to the high volatility of benzene and the variety of benzene emission sources, resulting in background concentrations not specific to the MWT fire [34]. As fires at recycling facilities are increasing in frequency in the US [4], these data suggest monitoring HCN concentrations at higher sensitivity may be a viable strategy to investigate the spatial distribution of VOCs released from the incidence site. Together, these data highlight the ability of this approach to detect and evaluate ambient concentrations of VOC in communities and highlight the increased sensitivity of the PTR-TOF over traditionally used field-deployable equipment.

In addition to benzene and HCN, plastic combustion also releases high concentrations of other potentially toxic VOCs [9]. However, predicting the identity of all potential combustion products from such variable, non-specific substrate mixtures is challenging. Here, we demonstrate that coupling NTA with the highly sensitive PTR-MS is a rapid and viable approach to generate a comprehensive list of the emitted VOCs following the combustion of variable substances. Using this approach, we detected 46 VOCs that were above local background concentrations, including benzene and HCN, across sampling sites within and bordering the mandated evacuation zone. We continually detected the largest number of total and unique VOCs at the sampling site closest to the MWT fire (Site 1), approximately 0.1 miles away. Across both site #1 visits, the identity and estimated average concentration of detected VOCs varied, illustrating the variability in VOC emissions from fires and supporting the need for frequent air monitoring throughout the duration of disasters. The number of total and unique VOCs across the Site 2 visits were consistently fewer than those detected across the site #1 visits. These data suggest that the 0.5 mile radius evacuation mandate may have been successful in decreasing the exposure of members of the local community to potentially toxic chemicals. However, it is also important to highlight that the decreased number of total and unique VOCs detected across the site #2 visits also coincided with a sampling time 6–8.5 h after the initial Site 1 Visit 1. Although still elevated in comparison to the number of total and unique VOCs detected across the site #2 visits, there was also a decrease in the number of total and unique VOCs detected at Site 1 Visit 2, approximately 8 h after the initial Site 1 Visit 1 stationary sampling. Thus, this information together may suggest the decreased number of VOCs detected at the Site 2 visits may also be a result of the overall reduction in VOC emissions from the MWT fire. Future studies conducting mobile air monitoring could improve this limitation by enabling data collection at Site 1 and Site 2 within closer time frames to help better investigate the relationship between VOC detection and proximity to the incidence site with potentially fewer confounding factors due to the status of the active fire. Notably, the EPA did not detect any of these 46 VOC on the same day of our monitoring, across any of their monitoring sites. These findings align with the growing field of evidence suggesting NTA approaches can improve broad chemical identification in disaster situations [1, 5].

The ability to generate a comprehensive hazard assessment of the broad list of chemicals emitted following a disaster is another important advantage of NTA approaches. Here, we demonstrate that the Hazard Comparison Module can be used to rapidly identify the potential acute human health hazards of each detected VOC [21, 22]. In brief, the Hazard Comparison Module enables users to identify chemical and hazard information to evaluate the potential health effects of chemicals. Through examining the 46 VOCs with estimated average concentrations higher than the local background concentrations, eye and skin irritation had the highest number of VOCs classified as “very high” – “high” hazard. A smaller number of VOCs were also associated with “acute dermal toxicity”, further suggesting exposed skin as a vulnerable target of the emitted VOCs from the MWT fire. Interestingly, while all detected concentrations were within safe limits, skin irritation was noted by several residents. As these safety limits were established for single chemical exposures, this discrepancy highlights the need for the investigation of chemical mixture exposure effects on human health and/or highlights the importance of including susceptible groups into these assessment studies. Notably, eye and skin irritation were also among the common symptoms reported by the residents in East Palestine following the Ohio train derailment [14]. Future studies investigating the health outcomes of the first responders and residents, such as that which occurred following the East Palestine train derailment [35], would provide valuable, complimentary data to the hazard assessment performed in this study. Moreover, a similar assessment of the health outcomes of the Richmond residents and first responders, within and bordering the evacuation zone, could be used to improve our understanding of the health risk of exposure to these VOCs for susceptible individuals, including pregnant women, children, and the elderly.

In combination with hazard assessment, the rapid identification of the exposure level of chemicals released during disaster scenarios is important for risk characterization, enabling responses that can improve the protection of first responders and the local community [1, 22, 36]. Thus, we estimated the exposure concentrations and emission relationships of the detected VOCs to support future risk assessment. All detected VOCs had elevated, but still considered safe, estimated average exposure concentrations. Of the 46 VOCs detected across the sample sites, 5 had estimated detection concentrations at least 1.8-fold higher than background concentrations during at least one site visit. Notably, certain VOCs in this group, such as C_2_H_6_N_2_, had fold changes consistently over 100-fold higher than background levels. In addition, multiple VOCs in this group (HCN, formic acid, acetone, propanal, and propylene oxide) were classified as either a “very high” or “high” hazard for one or more of the human health effects evaluated in the hazard assessment. 3 of these 5 elevated compounds, HCN, C_3_H_6_O, and CH_2_O_2_, have also been previously associated with plastic combustion [7, 27, 37–39]. While these highly elevated levels of potentially hazardous VOCs suggest they may be significant contributors of any observed adverse health effects, it is also important to consider the total exposure burden to improve hazard assessment. Therefore, we evaluated the spatial and temporal emission relationships of the 46 elevated VOCs and identified 5 large groups of at least 4 VOCs that were significantly, positively correlated. These data highlight VOCs that were likely emitted together, providing helpful information for future studies evaluating the exposure effects and risk assessment of real-world mixtures.

As no study is without limitations, we would like to acknowledge a few limitations specific to this investigation. First, we applied a default reaction rate to quantify compounds that we did not have calibration standards for. This approach (i.e., nontargeted analysis) can introduce uncertainty in absolute quantification, as reported by others [25, 40]. Second, although the PTR-TOF 4000 uses “soft” ionization technology, fragmentation of larger VOCs into products that can artificially inflate smaller VOC detection levels can occur due to the ionization procedure and other factors [41]. These additional contributing factors can be the specific identity of the parent VOC and/or atmospheric conditions related to time-of-day and seasonal conditions, as well as the extent of urbanization of the sampling location. Given these uncertainties, we have focused on relative abundance of VOCs in comparison to background levels (i.e., site #0, approximately 40 miles away from the incidence site). This is a useful approach to inform complex VOC mixtures when external calibration, known k rates, and determination of fragmentation are not possible or available, particularly in the case of rapid emergency responses. Third, there are additional tools for resolving VOCs at even higher resolution [40]. However, the instrumentation applied in this study (PTR-TOF 4000) still offers high sensitivity and resolution (>4000) feasible for conducting field studies in a mobile platform. This mobile application of the PTR-TOF 4000 is important, as it permits rapid data collection that is essential when responding to environmental emergency events.

Overall, the large and persistent MWT warehouse fire in Richmond, IN, prompted the need to conduct air monitoring measurements to identify the emitted air pollutants and assess the potential human health risk to the local community. We set out to complement the monitoring efforts initiated by the EPA through stationary air monitoring within and bordering the mandated evacuation zone. By coupling together NTA with the highly sensitive PTR-MS, we generated a comprehensive list of 46 VOCs elevated across sampling sites in Richmond, IN. By leveraging the EPA Hazard Comparison Module, we also generated a hazard assessment of the VOCs to identify most likely the human health effects from exposure to these compounds. Lastly, we determined chemical exposure levels and identified significant VOC emission relationships to support future risk assessment of the VOCs associated with the MWT warehouse fire. Together, these findings support the applicability of NTA coupled with hazard assessment as a valuable tool to identify unknown chemicals and their hazard potential in disaster scenarios.

## Supplementary information

Supplementary Method

Supplymentary Tables

## Data Availability

The data generated and analyzed for this study is provided in the supplemental information. Please contact corresponding author for any additional details at nmjohnson@tamu.edu.
