# Supplementary material for: The application of PTR-MS and non-targeted analysis to characterize VOCs emitted from a plastic recycling facility fire
Source: J Expo Sci Environ Epidemiol. 2024 May 6;35(2):149–56. doi: 10.1038/s41370-024-00681-y (PMC12009732; doi:10.1038/s41370-024-00681-y)
Supplement: Supplementary file 1 — Supplementary Method [file 41370_2024_681_MOESM1_ESM.pdf]

Supplementary Method S1: Proton transfer reaction time-of-flight reaction (PTR-ToF) mass spectrometer operation.

The “soft” ionization technology of PTR-ToF detects the identity of parent volatile organic compounds (VOCs) with minimal fragmentation of the ion. The Ionicon, Inc. 4000 time-of-flight mass spectrometer has a minimal mass resolution of 4,000 and can separate ions that have the same nominal mass but different combinations of C, H, O, and heteroatoms. The PTR-ToF was operated in one mode, the traditional hydronium mode. Ionization of the VOCs is achieved via a proton transfer reaction:

$$H_3^+O + X \rightarrow XH^+ + H_2O$$

Here, X is the analyte of interest. This mode is sensitive to species that have a higher proton affinity than water, which includes a wide array of atmospheric VOCs but crucially does not include small aliphatic molecules like methane and ethane that would otherwise dominate the signal.

At the start of the sampling day, zero air was “sampled” using the instrument to determine the instrument baseline. Additionally, a multi-point calibration was conducted at the beginning of the sampling day by diluting a mixture of calibrant gases with zero air (**Supplementary Table S6**).
